# Multimodal Treatment of a Canine Lingual Melanoma Using a Combination of Immunotherapy and a Tyrosine Kinase Inhibitors

**DOI:** 10.3390/vetsci9020054

**Published:** 2022-01-29

**Authors:** Alexander Berry, Alison Hayes, Luca Schiavo, Jane Dobson

**Affiliations:** Queen’s Veterinary School Hospital, University of Cambridge, Cambridge CB3 0ES, UK; ab2148@cam.ac.uk (A.B.); ls862@cam.ac.uk (L.S.); jmd1000@cam.ac.uk (J.D.)

**Keywords:** lingual malignant melanoma, immunotherapy, tyrosine kinase inhibitor, TKI

## Abstract

A 9-year-old female neutered Miniature Schnauzer was diagnosed with a lingual malignant melanoma on the basis of incisional biopsy and histopathology. The patient was initially given a guarded prognosis of a few months’ survival as surgical treatment options were declined by the owner. In order to control the disease a combination treatment of immunotherapy and tyrosine kinase inhibitors was initiated. The mass showed a marked and sustained reduction in size, whilst preserving quality of life for the patient, with a survival at the time of writing of 15 months since diagnosis. This experience suggests that combination therapy for oral malignant melanoma using immunotherapy and tyrosine kinase inhibitors may be successful in some patients and warrants further investigation.

## 1. Introduction

Malignant melanoma is the most commonly diagnosed canine oral tumour [1,2], representing approximately 1% of diagnosed canine neoplasia [3] and 33% of canine oral tumours [4]. Lingual masses are relatively uncommon in the dog, the majority being either non-neoplastic or benign in nature, such as those associated with inflammation of the tongue (glossitis), squamous papilloma and plasma cell tumour. Malignant melanoma has been reported to be the most common lingual neoplasia, representing 10–12% of lingual masses [5,6], though others reported squamous cell carcinoma to be more common than melanoma [7].

Canine melanocytic tumours can have a range of biological behaviour [8] which can be difficult to predict. Oral melanomas are generally reported to have a poor prognosis, with a metastatic rate of 53–67%, and a reported survival time of only 1 to 4 months without treatment, with most animals euthanised due to either progression of local disease, or evidence of gross metastasis [3,9,10,11]. Aggressive surgical excision with or without adjunctive radiotherapy has traditionally been regarded as the best approach to treatment of local disease, with survival times following surgery varying widely from 3 to 45 months [9,12,13]. Malignant melanoma is regarded as refractory to chemotherapy [14,15]. Stage I tumours, which are <2 cm in size and show no evidence of local metastasis, are reported to have the best prognosis, and following surgery a median survival time (MST) of 17–18 months has been reported [16], whilst more advanced tumours are associated with poorer survival times.

Other tumour characteristics have also been investigated for their prognostic utility. Mitotic index (MI) has been shown to be strongly correlated with prognosis, with melanotic tumours with an MI <3 associated with a significantly longer remission [17]. The degree of anisokaryosis has also been reported to be associated with shorter survival times in cutaneous melanomas [18], and in oral melanomas has been shown to be predictive of malignant behaviour and prognosis [11]. However, other studies have not found nuclear atypia to be of prognostic value, and so the picture remains unclear [19,20]. Similarly, the degree of pigmentation of oral melanomas has also not been found to be correlated with clinical outcome in several studies [3,12,17,19]. However, one study reported that a high degree of pigmentation is associated with longer survival, but that the absence of pigmentation was not associated with poor outcome [11]. The presence of ulceration of the lesion has not been found to be of utility in predicting remission length or survival time for oral melanomas [17], though the presence of intralesional necrosis has been found to be of prognostic value [11].

The relatively low incidence of lingual melanomas has led to a paucity of prognostic data for these tumours specifically. A study of 11 lingual melanomas reported a lower metastatic rate than other oral melanomas, of 38%. The same study also reported a MST following surgery of 241 days, though this was significantly affected by the presence of metastasis, with dogs without evidence of metastatic disease having a MST 3 times longer than those with metastases [7]. To the authors’ knowledge there is no specific data regarding survival time for patients with lingual melanoma managed medically or those receiving no treatment.

## 2. Case Presentation

A 9-year-old female neutered Miniature Schnauzer was presented to the oncology service at the Queens Veterinary School Hospital, University Cambridge (QVSH), Cambridge, UK, after an incisional biopsy by the referring veterinary surgeon confirmed a lingual melanoma.

The mass was reported on histopathology to be a poorly demarcated, non-encapsulated densely cellular neoplasm arising in the superficial submucosa of the tongue, and extending into the skeletal muscle of the tongue on its deep margin and to an ulcerated epithelial surface superficially. Neoplastic cells were described as polygonal with distinct borders and moderate pale eosinophilic to vacuolated cytoplasm, forming solid clusters, aggregates or vague streams within the pre-existing stroma of the tongue. There were approximately 10% of cells containing fine brown pigmented granules consistent with melanin. There was moderate anisocytosis and anisokaryosis, with a mitotic count of 16 mitosis per 10 high power fields, including some atypical forms. Immunohistochemistry and *c-kit* analysis were not performed, as diagnosis was achieved with standard histolopathological examination.

The mass had been identified during intubation for surgery to remove multiple suspected cutaneous mast cell tumours (MCTs), but these had subsequently not been removed due to the perceived poor prognosis of the lingual mass. At the time of presentation, the owners did not report any clinical signs related to the lingual mass, with the dog eating well and no dysphagia or sialorrhoea noticed.

**Figure A1:**
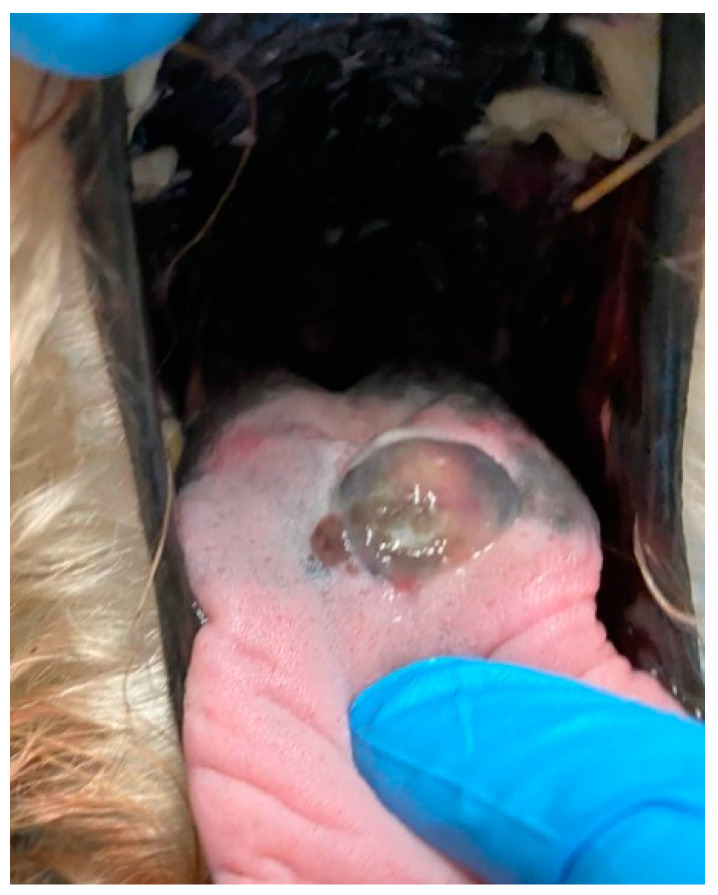
The mass at presentation to the QVSH.

On examination, an approximately 2cm in diameter, raised and pigmented mass was located on the caudal-dorsal aspect of the left side of the tongue (Figure A1). Submandibular lymph nodes were palpated as within normal limits, however full staging including thoracic imaging and lymph node cytology was declined by the owners at the time of first diagnosis. Subsequent thoracic radiography, after 7 months of treatment, did not demonstrate any evidence of metastatic disease. On the basis of tumour size and lack of evidence of lymph node metastasis, the tumour was deemed to be WHO T stage 2 (Table 1), however it could not be ruled out that it was in fact stage 3 or 4, as lymph node size is poorly predictive of metastatic disease [10]. Due to the location and size of the mass, neither surgical removal nor radiotherapy were deemed viable treatment options due to the likely morbidity associated with treatment to the tongue in this location. After discussion with the owners a course of immunotherapy with xenogenic human tyrosinase DNA canine melanoma vaccination (Oncept, Boehringer Ingelheim) was commenced, with adjunctive use of the tyrosine kinase inhibitor (TKI) masitinib (Masivet, AB Science), with the aim of slowing or preventing tumour growth in order to preserve quality of life.

**Figure A2:**
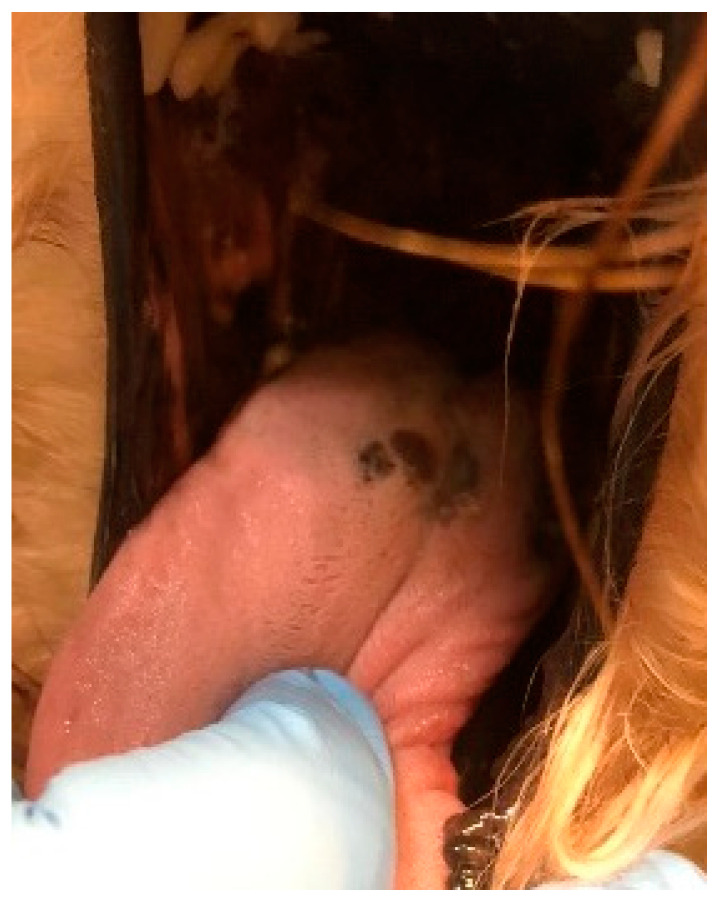
Appearance of the mass at the end of immunotherapy.

A standard protocol of intramuscular immunotherapy every 14 days for 4 doses was followed, and masitinib was given concurrently at 12.5 mg/kg orally for 6 days out of seven. Routine biochemistry and haematology were performed at approximately monthly intervals throughout. At the time of the third immunotherapy dose, 4 weeks after therapy was initiated, the tumour had markedly reduced in size, and measured approximately 1cm in diameter. According to RECIST criteria [22], this constituted a partial response to treatment. Treatment was well tolerated, though moderate elevation in ALT and ALP were apparent on serum biochemistry. At the time of the final immunotherapy dose of the primary course, a further reduction in size of the mass was noted, which measured approximately 0.5 cm in diameter and was no longer as raised above the surface of the tongue (Figure A2). Biochemistry showed a persistent moderate elevation in ALT, though reduced from the previous value.

**Figure A3:**
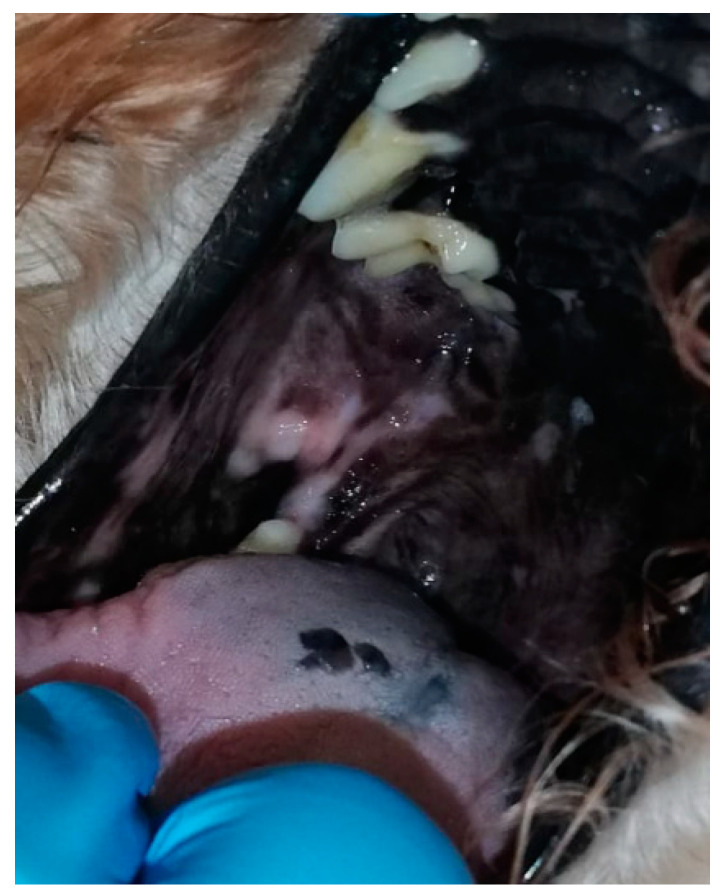
Appearance of the mass 3 months after completion of immunotherapy.

After completion of the immunotherapy course, examination was repeated at monthly intervals and masitinib therapy was continued. Four months after treatment was commenced progressive disease was noted (time to progression/TTP—4 months), with an increase in size and prominence of the lingual lesion (Figure A3).

As this was less than 6 months after the end of the primary course of immunotherapy, a booster dose was not given. It was considered likely that the melanoma had become refractory to masitinib, and this was substituted with oral toceranib (Palladia, Pfizer Animal Health) 2.91 mg/kg given on alternate days.

Following initiation of toceranib the mass again showed partial response to treatment, with marked reduction in size and prominence. At the recheck 6 months after finishing the initial course of immunotherapy the mass was much reduced in size, and a booster dose of immunotherapy was given. Approximately 3 months later the dog developed pancreatitis and toceranib treatment was paused for approximately 2 weeks. The dog recovered uneventfully.

**Figure A4:**
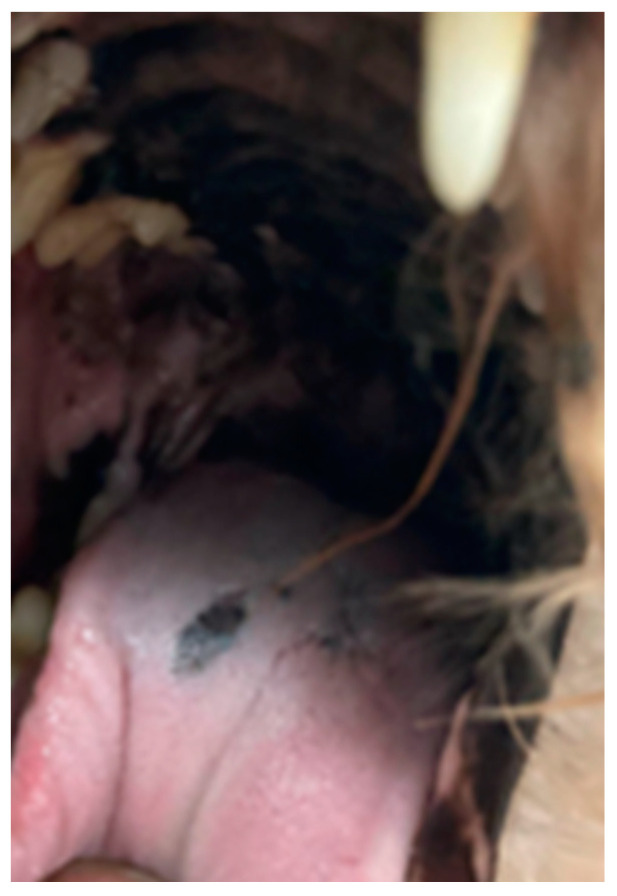
Appearance of the mass at final re-visit, 15 months after diagnosis.

At time of last examination, 13 months after initiation of treatment, the lingual mass was only just visible (Figure A4), and treatment with toceranib continued to be well tolerated, with unremarkable haematology and biochemistry. Subject to supply and continued response, the immunotherapy is planned to continue at regular sixth month intervals.

## 3. Discussion

A lack of published information on lingual melanomas makes it difficult to provide an accurate prognosis to owners. However, the mitotic index of this tumour was high at 16 mitoses per 10 high power fields was high, and potentially a poor prognostic indicator for survival time [17]. Due to the high metastatic rate of malignant melanomas and perceived poor prognosis, the owners declined surgical intervention in this case, which would have required total glossectomy. Whilst this has been reported with some success in the dog, post-operative requirement for feeding via gastrotomy or oesophagostomy tubes, difficulty in prehending food and relatively long recovery periods are also reported [7,23,24]. Radiotherapy of the lesion in this case would have involved irradiating the tongue base and risked compromising the viability of the entire tongue and was consequently declined by the owners. Whilst chemotherapeutic options have been previously explored in cases of canine oral and lingual melanoma, no effective systemic treatments are currently available.

Adjunctive treatment of canine oral melanoma with DNA vaccination has been reported to have variable efficacy, with initial studies indicating good clinical response in approximately 40% of dogs and increasing MST to 389 days [25], though other studies have suggested that it did not increase disease free time or MST [26,27].

Combined treatment with immunotherapy and oncogene targeting therapy is reported to have some benefit in human mucosal melanoma treatment, with ongoing research into this combination therapy [28,29]. In humans the *c-kit* gene, which encodes a tyrosine kinase receptor, has been demonstrated to be expressed, overexpressed and/or mutated in some mucocutaneous melanomas [30], and also shown to be expressed in some canine oral melanomas [31], where its expression has been shown to be associated with significantly longer survival times than those tumours without *c-kit* expression. It has been suggested that *c-kit* may be down regulated as invasive growth patterns emerge. [31]. More routine use of PCR to undertake c-kit mutation analysis in cases of oral melanomas may assist in more empirical treatment selection, and was not undertaken for the case presented here.

The small molecule inhibitors masitinib and toceranib are licensed to treat non-resectable Patnaik grade II or III canine cutaneous mast cell tumours. They have also been reported to be of benefit in other tumour types including melanoma [9], apocrine gland anal sac adenocarcinoma [32], thyroid carcinoma [33] and epitheliotrophic lymphoma [34]. Drug targets are the receptor tyrosine kinase family of transmembrane proteins including stem cell factor receptor (*c-kit*), platelet derived growth factors (PDGFRs), lymphocyte specific protein kinase (Lck), focal adhesion kinase (Fak) in the case of masitinib [9] and additionally in the case of toceranib: vascular endothelial growth factor receptor (VEGFR), stem cell factor (SCF), colony stimulating factor-1 (CSF-1), glial derived neurotrophic factor (GDNF), and FMS related tyrosine kinase 3 ligand (FLT3L) [35].

Given the documented expression of *c-kit*, VEGFR and PDGFR [36,37,38] in oral melanomas researchers have explored the therapeutic use of TKIs in canine melanoma [9]. Masitinib has previously been reported to be of very variable efficacy in treatment of oral melanomas and was deemed not an appropriate sole-agent option for treatment of advanced malignant melanoma in a cohort of dogs [9]. Treatment of canine oral melanoma with toceranib may offer benefits due its wider range of molecular targets, and has been previously reported to have some efficacy in a single case of advanced metastatic oral melanoma, however survival time was only 54 days [39].

## 4. Conclusions

To the authors’ knowledge this is the first case report detailing a demonstrable and sustained response of lingual melanoma to a combination of immunotherapy and tyrosine kinase inhibitor. The case reported here has now survived 15 months from the time of first diagnosis of lingual melanoma, which is significantly longer than the reported MST of 119 days for dogs treated with masitinib [9]. Furthermore, treatment has been well tolerated and preserved quality of life for the patient throughout. Although this is only a single case report, the demonstrable and sustained response of this lingual melanoma to the combination of immunotherapy with TKI’s suggests that this approach to the management of canine oral malignant melanoma may be of value in other cases and is worthy of further investigation.

## Figures and Tables

**Table 1:** WHO criteria for staging canine oral melanoma on the basis of size and/or presence of metastatic disease [21].

Stage	Tumour Size	Presence of Metastatic Disease
I	≤2 cm diameter	No
II	2 < 4 cm diameter	No
III	> or = 4 cm diameter	Evidence of lymph node metastasis, irrespective of size of primary tumour
IV	Any	Evidence of distant metastasis

## Data Availability

The data presented in this study are available on request from the corresponding author.
